# Epstein–Barr Virus Susceptibility in Activated PI3Kδ Syndrome (APDS) Immunodeficiency

**DOI:** 10.3389/fimmu.2017.02005

**Published:** 2018-01-16

**Authors:** Jean-Marie Carpier, Carrie L. Lucas

**Affiliations:** ^1^Immunobiology Department, Yale University School of Medicine, New Haven, CT, United States

**Keywords:** Activated PI3Kδ Syndrome, PASLI, PI3K/AKT/mTOR, Epstein–Barr virus, immunodeficiency, B cell, T cell

## Abstract

Activated PI3Kδ Syndrome (APDS) is an inherited immune disorder caused by heterozygous, gain-of-function mutations in the genes encoding the phosphoinositide 3-kinase delta (PI3Kδ) subunits p110δ or p85δ. This recently described primary immunodeficiency disease (PID) is characterized by recurrent sinopulmonary infections, lymphoproliferation, and susceptibility to herpesviruses, with Epstein–Barr virus (EBV) infection being most notable. A broad range of PIDs having disparate, molecularly defined genetic etiology can cause susceptibility to EBV, lymphoproliferative disease, and lymphoma. Historically, PID patients with loss-of-function mutations causing defective cell-mediated cytotoxicity or antigen receptor signaling were found to be highly susceptible to pathological EBV infection. By contrast, the gain of function in PI3K signaling observed in APDS patients paradoxically renders these patients susceptible to EBV, though the underlying mechanisms are incompletely understood. At a cellular level, APDS patients exhibit deranged B lymphocyte development and defects in class switch recombination, which generally lead to defective immunoglobulin production. Moreover, APDS patients also demonstrate an abnormal skewing of T cells toward terminal effectors with short telomeres and senescence markers. Here, we review APDS with a particular focus on how the altered lymphocyte biology in these patients may confer EBV susceptibility.

## Introduction

Epstein–Barr virus (EBV) is a gammaherpesvirus carried by ~95% of the world population. EBV has a tropism for oronasopharyngeal epithelial cells (site of lytic replication) and B lymphocytes (reservoir of latent virus) and is well controlled throughout life in most people. However, immunocompromised patients often show persistent EBV viremia, putting them at risk for B-cell transformation due to viral oncogenes. Indeed, the virus was first identified in a Burkitt’s lymphoma in the 1960s (1) and is also associated with nasopharyngeal (2, 3) and gastric (4–7) cancer. Thus, inherited gene defects causing primary immunodeficiency diseases (PIDs) are often associated with recurrent or persistent EBV infections and related malignancies, and unraveling the genetic and molecular mechanisms underlying PIDs has led to better knowledge of the cellular and molecular components of the immune system that control herpesviruses. Here, we review the features of the recently described PID called **A**ctivated **P**I3K**δ S**yndrome (APDS) and discuss the immunological abnormalities that may confer susceptibility to EBV and elucidate the cellular and molecular immune mechanisms normally controlling EBV.

The Class IA phosphoinositide 3-kinase delta (PI3Kδ) complex is recruited to phosphotyrosines and catalyzes the phosphorylation of phosphatidylinositol-4,5-bisphosphate to generate phosphatidylinositol-(3,4,5)-trisphosphate (PIP_3_) that acts as a second messenger recruiting downstream signaling molecules. As a negative regulator of this signaling, the phosphatase PTEN can reverse this reaction and reduce levels of PIP_3_. PI3Kδ is a heterodimer of the p110δ catalytic subunit and the p85α, p55α, or p50α regulatory subunit and is known to play a major role in cell survival, cell growth, and cell-cycle entry through downstream mediators including AKT and mTORC1 (8). Loss of PI3Kδ catalytic activity has been described in a single PID patient with severe disease, but EBV susceptibility was not reported (9). Gain-of-function (GoF) mutations in the *PIK3CD* or *PIK3R1* gene encoding p110δ or p85α, respectively, have been identified by us and others in PID patients with a disorder now known as PASLI Disease (**P**I3Kδ-**A**ctivating mutation causing **S**enescent T cells, **L**ymphadenopathy, and **I**mmunodeficiency), or APDS for short. In the following sections, we will briefly review the discovery of APDS and its genetic and molecular basis, the clinical and immunological features of APDS, and possible contributors to poor control of EBV in APDS patients.

## Genetic and Molecular Basis of APDS

Activated PI3Kδ Syndrome and causative *PIK3CD* mutations were initially described in two reports with a total of 26 patients in 14 unrelated families (10, 11). Prior to these initial reports, there had been one description of the most frequent mutation in *PIK3CD* (causing E1021K p110δ) in a single individual being studied for B-cell immunodeficiency, but no causative relationship was established (12). Shortly after discovery of APDS and underlying *PIK3CD* mutations, two additional reports with eight patients from six unrelated families with similar clinical findings described splice site mutations in *PIK3R1* as a second genetic cause for APDS (13, 14). Thus, APDS1 (or PASLI-CD) has been established to denote patients with *PIK3CD* mutations, and APDS2 (or PASLI-R1) denotes those with *PIK3R1* mutations. Another more recent phenocopy of APDS has been called APDS-like (APDS-L) and is caused by loss-of-function *PTEN* mutations (15, 16). Since the description of APDS in 2013, approximately 214 patients have been described with a spectrum of clinical features described below (10, 11, 13–41).

**Figure 1:**
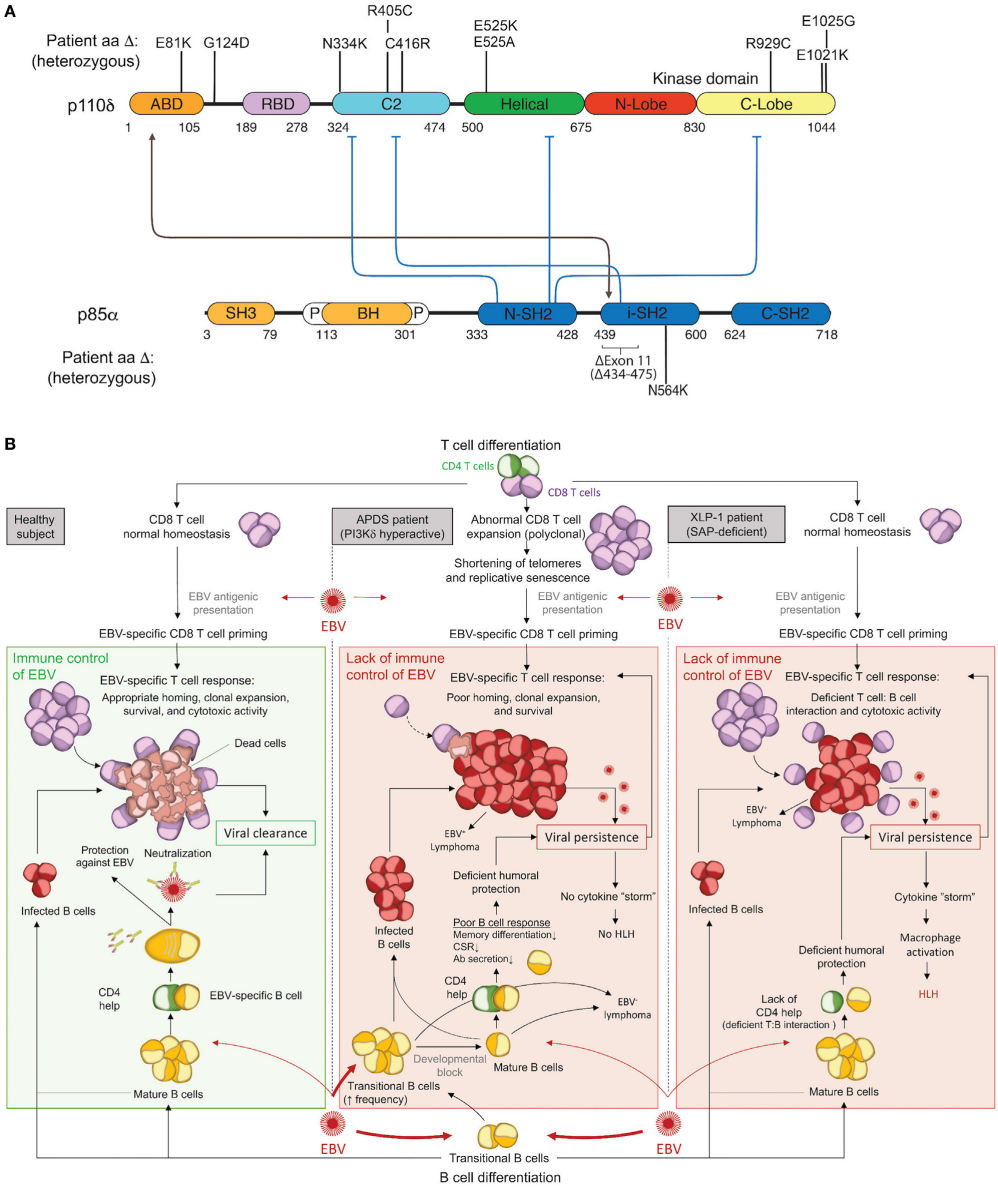
Activated PI3Kδ Syndrome (APDS) GoF mutations in the PI3Kδ complex and associated immune dysfunction responsible for Epstein–Barr virus (EBV) susceptibility. **(A)** Schematic representation of p110δ and p85α protein domains and APDS mutations reported in patients. The black line depicts the stabilizing interaction, and the blue lines show the inhibitory contacts within the PI3Kδ complex. ABD, adaptor-binding domain; BH, breakpoint-cluster region homology domain; P, proline-rich region; SH, SRC-homology domain; N, amino-terminal; i, inter; C, carboxy-terminal. **(B)** Schematic representation of the current understanding for the immune control of EBV in healthy subjects (left) and proposed hypothesis for EBV susceptibility in APDS (middle) and XLP1 (right) patients. APDS mutations cause abnormal polyclonal expansion of CD8 T cells that become senescent. Senescent CD8 T cells show an impaired EBV-specific response due to limited homing, expansion, and survival. In conjunction with CD8 T-cell defects, APDS patients exhibit an elevated frequency of transitional B cells, a major cell type for cell entry of EBV, and have defective humoral immunity that may further contribute to EBV susceptibility. In comparison, XLP1 patients, who are susceptible to EBV and develop HLH, are deficient in the SAP adaptor and exhibit defective EBV-specific T cell: B-cell interactions, causing a lack of CD4 help and a failure of CD8 T-cell cytotoxicity. As opposed to APDS, viral persistence in XLP1 patients causes a recurring stimulation/expansion of EBV-specific CD8 T cells and results in a cytokine storm underlying hemophagocytic lymphohistiocytosis (HLH). Antibodies depiction: taken from SMART (Servier Medical Art) licensed under a Creative Commons Attribution 3.0 Unported License.

The PI3Kδ complex forms when p110δ and p85α bind at a 1:1 ratio. This constitutive complex remains stable due to tight binding interactions between the adaptor-binding domain (ABD) of p110δ and the inter-SH2 domain of p85α. To date, all activating APDS mutations affecting p110δ (E81K, G124D, N334K, R405C, C416R, E525K, E525A, R929C, E1021K, E1025G) and p85α (delE11, N564K) have been found or are expected to maintain some level of protein–protein interaction to form a hyperactive PI3Kδ complex, as free p110δ or p85α is unstable and would likely be degraded (Figure 1A). Each evaluated mutant has been found to hyperactivate signaling by disrupting inter- or intra-molecular inhibitory contacts, as observed for tumor-associated GoF mutations in the related *PIK3CA* (Figure 1A) (42, 43).

## Clinical and Cellular Features of APDS

The clinical spectrum of APDS1, APDS2, and APDS-L is largely overlapping and consists mostly of immunological abnormalities (Table 1), although growth retardation has also been reported APDS2 and, less frequently, APDS1 (10, 12–14, 17, 21, 24, 26, 27, 29–33, 37). Recurrent upper and lower respiratory tract infections are the most common clinical features affecting 98% of APDS patients and often resulting in progressive airway damage. APDS is associated with lymphoproliferative disease (71%), which commonly presents as lymphoid hyperplasia, splenomegaly, and/or lymphadenopathy. Autoinflammatory disease also occurs in 29% of cases. Importantly, recurrent infection with herpesviruses, such as EBV or cytomegalovirus (CMV), is observed in about 47% of cases but has not been associated with hemophagocytic lymphohistiocytosis (HLH). We hypothesize that HLH does not occur in APDS patients because, as described below, hyperactive PI3K drives polyclonal T-cell senescence, which limits homing, expansion, and survival of EBV-specific T cells and thereby prevents the cytokine storm that causes HLH (Figure 1B). EBV infection is found in 30% of APDS patients and represents an important risk factor for the development of B-cell lymphoma (occurring in 20% of EBV-infected APDS patients). However, the occurrence of EBV-negative lymphomas has overall been reported as higher (19%) than EBV-positive lymphomas (6%), which likely reflects the oncogenic potential of hyperactive PI3K signaling. Thus, intrinsically hyperactive PI3K (rather than EBV infection) appears to be the more dominant driver of B-cell transformation in APDS.

**Table 1:** Summary of clinical and immunological features of APDS patients.

				Clinical features						Immunological features				
Reference	Gene	Mutation^a^	Number of patients	Respiratory infections^b^	Lympho-proliferation^c^	EBV viremia	Other herpesviruses	B lymphoma	EBV + B lymphoma	Increased immature/transitional B cells	Decreased IgA and/or IgG titers	Increased IgM titers	Defect in memory B cell^d^	Increased CD8 differentiation^e^
Jou et al. (12)	*PIK3CD*	E1021K	1	1/1	n.d.	n.d.	1/1 (VZV)	n.d.	n.d.	n.d.	1/1	1/1	n.d.	n.d.

Angulo et al. (10)	*PIK3CD*	E1021K	17	17/17	10/17	1/17	4/17	1/17	n.d.	14/16	10/11	14/17	8/16	5/5

Lucas et al. (11)	*PIK3CD*	E1021K	3	3/3	3/3	3/3	1/2	1/3	1/3	3/3	2/3	2/3	2/2	2/2

	*PIK3CD*	E525K	5	5/5	3/5	5/5	4/5	1/5	1/5	5/5	3/5	0/5	3/5	1/1

	*PIK3CD*	N334K	1	1/1	1/1	1/1	0/1	0/1	0/1	1/1	1/1	1/1	1/1	1/1

Crank et al. (17)	*PIK3CD*	E1021K	1	1/1	1/1	0/1	0/1	1/1	0/1	1/1	1/1	1/1	n.d.	n.d.

	*PIK3CD*	C416R	2	2/2	2/2	1/2	1/2 (HSV)	2/2	0/2	2/2	1/2	2/2	n.d.	n.d.

Deau et al. (13)	*PIK3R1*	delE11	4	4/4	1/4	1/4	1/4 (CMV)	n.d.	n.d.	3/4	4/4	3/4	2/4	2/3

Kracker et al. (18)	*PIK3CD*	E1021K	8	8/8	6/8	0/8	0/8	2/8	0/8	0/1	5/8	7/8	2/2	n.d.

Lucas et al. (14)	*PIK3R1*	delE11	4	4/4	3/4	0/3	1/3 (CMV)	1/4	n.d.	n.d.	4/4	1/3	n.d.	Majority

Hartman et al. (19)	*PIK3CD*	E1021K	5	5/5	1/5	0/3	2/5 (HSV1, VZV)	n.d.	n.d.	n.d.	1/5	4/5	4/5	n.d.

Kannan et al. (20)	*PIK3CD*	E1021K	1	1/1	1/1	1/1	0/1	0/1	0/1	1/1	1/1	1/1	1/1	1/1

Lougaris et al. (21)	*PIK3R1*	delE11	4	4/4	4/4	n.d.	n.d.	n.d.	n.d.	2/2	4/4	4/4	3/3	n.d.

Elgizouli et al. (23)	*PIK3CD*	E1021K	5	5/5	5/5	1/5	1/5 (CMV)	0/5	0/5	2/4	5/5	1/5	2/4	n.d.

Elkaim et al. (24)	*PIK3R1*	delE11	36	36/36	22/36	8/36	6/35 (CMV), 2 (VZV)	10/36	1/36	14/15	27/35	18/31	11/19	10/10

Kuhlen et al. (29)	*PIK3R1*	delE11	1	1/1	1/1	0/1	1/1 (CMV)	n.d.	n.d.	n.d.	1/1	1/1	1/1	1/1

Martínez-Saavedra et al. (25)	*PIK3R1*	delE11	1	1/1	0/1	n.d.	n.d.	n.d.	n.d.	1/1	1/1	1/1	1/1	1/1

Olbrich et al. (26)	*PIK3R1*	delE11	2	1/2	2/2	2/2	2/2	n.d.	n.d.	1/1	2/2	2/2	2/2	1/1

Petrovski et al. (27)	*PIK3R1*	delE11	4	4/4	4/4	0/4	0/4	0/4	0/4	2/4	4/4	2/4	4/4	1/4

Rae et al. (28)	*PIK3CD*	E1021K	1	1/1	1/1	n.d.	n.d.	n.d.	n.d.	n.d.	n.d.	n.d.	1/1	1/1

Tsujita et al. (15)	*PIK3CD*	E1021K	2	2/2	2/2	0/2	1/2 (HSV)	0/2	0/2	2/3	2/2	0/2	2/2	n.d.

	*PIK3CD*	E525A	3	2/3	2/3	0/3	1/3 (Herpes zoster)	0/3	0/3	3/3	3/3	1/3	0/3	n.d.

Bravo García-Morato et al. (30)	*PIK3R1*	delE11	2	2/2	2/2	1/2	1/2 (herpetic lesions)	1/2	0/2	1/1	2/2	1/2	0/1	1/1

Chiriaco et al. (22)	*PIK3CD*	E1021K	1	1/1	1/1	1/1	0/1	0/1	0/1	1/1	1/1	1/1	0/1	1/1

Coulter et al. (31)	*PIK3CD*	E1021K or E525K	50 + 3	51/53	39/53	14/53	49% including EBV + (human herpesvirus 6, VZV, HSV)	7/53	3/53	24/32	21/49	38/50	17/30	17/18

Dulau et al. (35)	*PIK3CD*	E1021K	5	5/5	5/5	4/5	4/5 (CMV, HSV, VZV)	2/5	n.d.	4/5	3/5	4/5	5/5	n.d.

	*PIK3CD*	E525K	3	3/3	3/3	3/3	2/3 (CMV)	1/3	n.d.	3/3	2/3	1/3	2/3	n.d.

	*PIK3CD*	N334K	1	1/1	1/1	1/1	0/1	0/1	n.d.	1/1	1/1	1/1	0/1	n.d.

	*PIK3CD*	E1025G	1	1/1	1/1	1/1	1/1 (VZV)	0/1	n.d.	0/1	1/1	1/1	1/1	n.d.

Mettman et al. (41)	*PIK3CD*	E1021K	1	1/1	1/1	n.d.	n.d.	0/1	0/1	n.d.	0/1	1/1	1/1	n.d.

Goto et al. (40)	*PIK3CD*	E1021K	1	n.d.	1/1	1/1	1/1 (CMV)	0/1	0/1	1/1	1/1	1/1	1/1	1/1

Hauck et al. (37)	*PIK3R1*	delE11	3	3/3	2/3	1/3	0/3	1/3	1/3	0/2	2/3	2/3	n.d.	2/2

Wentink et al. (34)	*PIK3CD*	E1021K	9	9/9	3/9	2/9	n.d.	2/9	n.d.	Increased	5/11	5/11	Decreased	n.d.
		
	*PIK3CD*	E525K	1	1/1	1/1	0/1	n.d.	0/1	n.d.		0/1	0/1		n.d.
		
	*PIK3CD*	R929C	1	1/1	0/1	0/1	n.d.	0/1	n.d.		1/1	0/1		n.d.
		
	*PIK3R1*	N564K	1	1/1	0/1	0/1	n.d.	0/1	n.d.		0/1	0/1		n.d.
		
	*PIK3R1*	delE11	1	1/1	1/1	0/1	n.d.	0/1	n.d.		0/0	0/0		n.d.

Nademi et al. (36)	*PIK3CD*	E1021K	10	10/10	8/10	2/10	5/10	1/11	n.d.	n.d.	n.d.	n.d.	n.d.	n.d.
	
	*PIK3R1*	delE11	1	1/1	0/1	0/1	0/1		n.d.	n.d.	n.d.	n.d.	n.d.	n.d.

Takeda et al (33)	*PIK3CD*	G124D	2	2/2	2/2	2/2	2/2 (Herpes zoster, labialis)	1/2	1/2	1/1	2/2	2/2	0/1	1/1

	*PIK3CD*	E81K	1	1/1	1/1	1/1	0/0	1/1	n.d.	0/1	0/0	0/0	1/1	0/0

Heurtier et al. (32)	*PIK3CD*	E81K	1	1/1	1/1	n.d.	n.d.	n.d.	n.d.	1/1	1/1	0/1	1/1	1/1

	*PIK3CD*	G124D	2	2/2	2/2	n.d.	n.d.	n.d.	n.d.	1/1	2/2	1/2	2/2	2/2

Rae et al. (38)	*PIK3CD*	R405C	1	1/1	0/1	0/1	0/1	0/1	0/1	n.d.	1/1	0/1	1/1	0/1

Saettini et al. (39)	*PIK3CD*	E1021K	1	1/1	1/1	1/1	0/1	0/1	0/1	1/1	1/1	0/1	1/1	1/1

			**214**	**98.1%**	**70.9%**	**29.5%**	**32.10%**	**18.80%**	**5.80%**	**80.7%**	**68.1%**	**65.3%**	**65.4%**	**70.3%**

*^a^Frequencies of activating PI3Kδ mutations among APDS1 and APDS2 patients: E1021K, 58%; C416R, 1%; R405C, 0.5%; E525K, 6%; E525A, 1%; N334K, 1%; E81K, 1%; G124D, 2%; R929C, 0.5%; E1025G, 0.5%; delE11, 29%; N564K, 0.5%*.

*^b^Includes upper and lower respiratory tracts*.

*^c^Includes splenomegaly and lymphadenopathy*.

*^d^Assessment of cell counts, frequency or B-cell memory class switch*.

*^e^Frequencies of effector/memory cells, CD57 expression, telomere lengths*.

*n.d., not determined; CMV, cytomegalovirus; EBV, Epstein–Barr virus; HSV, herpes simplex virus; VZV, varicella zoster virus*.

The susceptibility to infections displayed by APDS patients is associated with deficiencies in both T and B lymphocyte function, a feature that categorizes APDS as a combined immunodeficiency (Table 1). B-cell compartment abnormalities have been universally described in both APDS1 and APDS2. B-cell lymphopenia is found in 74% of patients and may be due to a developmental defect at the transitional stage, as IgD^+^CD10^+^ B cells are consistently increased in APDS patient blood (81%). Additionally, humoral defects have been observed in the majority of APDS patients, leading to poor vaccine responses in some patients. Serum concentrations of IgM are increased in 65% of cases, while IgA and at least one IgG isotype are decreased (68%). This phenotype suggests a defect in class-switch recombination (CSR), and *in vitro* studies have not yet provided a clear conclusion about whether this defect arises predominantly from B-cell-intrinsic or -extrinsic effects of PI3Kδ hyperactivation (11, 17, 22, 44). Although immunodeficiency is a major feature of APDS, expansion of CD8 T cells is commonly observed (70%) and, together with CD4 lymphopenia, explains the inverted CD4:CD8 ratios found in the disease (71%). In addition, the constitutive activation of PI3K is also linked to the progressive differentiation of T cells toward effector memory and terminally differentiated (T_EMRA_) subtypes. Consistently, CD8 T cells from APDS patients exhibit normal degranulation activity (induced by anti-CD3 stimulation) and TNF/IFNγ production (11) with reduced secretion of IL-2, weak proliferative responses, and enhanced restimulation-induced cell death (RICD) (10, 11, 14, 22).

Thus, APDS is characterized by a complex spectrum of clinical, immunological, and cellular features. Elucidation of the genetic and molecular defects has improved diagnosis and care of APDS patients (45). Because of the recurrent sinopulmonary infections, antibiotics are often given prophylactically, and immunoglobulin replacement is commonly used, although recurrent infections have been reported despite this treatment (15, 20, 26). Chemo- and/or radiotherapy are often used for lymphomas, a major cause of death in APDS patients (about 62% of deaths) (11, 14, 17–19, 24, 30, 31, 37). Beyond the treatment of these specific symptoms, hematopoietic stem-cell transplantation has proven beneficial for restoration of immune function in 67% of APDS patients receiving this therapy, which requires availability of an HLA-compatible donor and is particularly risky in the setting of EBV infection (10, 14, 15, 18, 24, 31, 34, 36). Identification of the genetic and molecular etiology of APDS has also led to more specific treatments, such as the use of the mTORC1 inhibitor (rapamycin) (10, 11, 23, 24, 26, 28, 34, 40) and specific p110δ inhibitors, which are currently being evaluated for APDS treatment in clinical trials.

## EBV Susceptibility in APDS Patients

### B-Cell Dysfunction

Epstein–Barr virus is usually acquired during childhood and is asymptomatic throughout life, while primary infection in young adulthood can (in ~30–70% of cases) cause infectious mononucleosis (IM) (46). Although control of EBV infection by the immune system has been mainly attributed to CD8 T cells and to a lesser extent to NK cells, a role for humoral immunity in protecting from EBV infection has recently been reevaluated with a focus on IM patients (46–48). Although a neutralizing antibody response against several viral proteins such as gp350, a particularly immunogenic EBV protein, is detectable in these patients (47), the peak of this antibody response occurs after disappearance of IM symptoms and clearance of the virus, and this delay has been attributed to B-cell dysfunction in acutely infected patients (46). Several vaccination strategies have focused on the gp350 protein (49–51) since it acts as a major mediator for entry of EBV into B cells through its interaction with CD21 (52). Interestingly, vaccination using recombinant gp350 in phase-I and-II trials correlated with a gp350-specific antibody response and showed a protective effect in IM development but not in asymptomatic EBV infections (50, 51). Thus, the role of neutralizing antibodies in protecting B cells from infection and lowering the extent of infection during primary exposure can be considered in asymptomatic individuals and especially in children who might carry maternal EBV-specific antibodies. This protection might also be crucial to prevent disease upon reexposure to EBV. As such, the defects in B-cell development and function observed in APDS patients might help explain their increased susceptibility to EBV.

Changes in B-cell differentiation and intrinsic B-cell dysregulation may also be relevant contributors to EBV susceptibility in APDS. The nature of the B-cell compartment primarily infected by EBV has been a matter of debate, and it was first proposed that IgD^−^CD27^+^ memory B cells are the major entry point (53). However, *in vitro* observations as well as data from IM patients suggested that primary infection of B cells occurs in naïve IgD^+^CD27 cells, which then undergo differentiation in germinal center reactions, resulting in the emergence of class-switched memory B cells carrying EBV (54, 55). The observation that APDS patients exhibit an increased frequency of immature transitional CD10^+^ B cells and have a low frequency of memory CD27^+^ B cells (11) while remaining highly susceptible to EBV may support the possibility that EBV can also infect developing B cells. Indeed, several studies performed in mice have reported the ability of developing B cells to be infected by EBV (56) or the homologous γ-herpesvirus MHV68 (57, 58). The idea that transitional B cells might be a critical entry point and reservoir for EBV has been proposed before and fits with a model in which recurrent seeding of the developing B-cell compartment with EBV virions promotes establishment of long-term B-cell infection (57). In agreement with this hypothesis, depletion of transitional B cells in mice reduces EBV in the mature B-cell compartment (58). Therefore, it is possible that persistent EBV infection is facilitated in APDS patients by the predominant transitional B-cell compartment that would provide a pathologically increased reservoir of EBV, although additional studies are required to evaluate this hypothesis.

The EBV latency proteins LMP2a and LMP1 are thought to be key players in hijacking B-cell maturation by EBV since they mimic B-cell receptor and CD40 signaling, respectively (59, 60). LMP1 in particular is sufficient to transform several cell types, activates PI3K signaling, and promotes B-cell survival, growth, and proliferation programs (59–61). As p110δ is the main Class IA PI3K isoform expressed in EBV-positive B-cell lymphomas, this isoform might be a major target for LMP1 (62), and EBV-driven lymphomas in APDS may thus be facilitated in B cells expressing hyperactive forms of PI3Kδ. Moreover, several studies have demonstrated that PI3K inhibition reduces EBV reactivation (59, 63, 64), suggesting that the increased PI3Kδ activity displayed by APDS patients would favor a constitutive lytic program and may contribute to persistent viremia.

Thus, APDS patients harbor abnormal B cells that likely promote EBV susceptibility through several mechanisms. These may include, among others, poor anti-EBV antibody responses, increased transitional B cells serving as an EBV reservoir, and heightened cell-intrinsic PI3K signaling that may promote EBV-driven B-cell transformation and/or EBV reactivation.

### T-Cell Dysfunction

T lymphocytes are a crucial immune cell type for control of EBV infection (65, 66). Substantial expansion of EBV-specific CD8 T cells has been observed in IM patients (67), and EBV control in healthy carriers has been correlated with the presence of functional EBV-specific CD8 T cells (68). However, the major arguments supporting a functional role for CD8 T cells in controlling EBV *in vivo* come from immunocompromised patients. Indeed, post-transplant lymphoproliferative disease (PTLD) is an important clinical concern in immunosuppressed transplant patients. In these patients, PTLD is caused by EBV-driven B-cell expansion and can be overcome by infusing EBV-specific cytotoxic T cells (69–72). Moreover, immunodeficiency syndromes, particularly HLH and X-linked lymphoproliferative diseases, have also provided valuable lessons and advanced our understanding of the role for CD8 T cells in EBV immunity (73, 74).

Monogenic causes of EBV-associated HLH have demonstrated that defective cytotoxicity machinery most commonly underlies disease (66, 75). However, these more general defects are not present in APDS patients, highlighting a more nuanced mechanism conferring EBV susceptibility when PI3K signaling is hyperactive. XLP1 patients deficient in the signaling lymphocytic activation molecule-associated protein (SAP) adaptor exhibit a very specific vulnerability to EBV viremia, and uncovering the genetic mutations responsible for disease contributed to defining crucial and non-redundant molecular pathways for EBV control by cytotoxic cells (76–79). Indeed, mutations in *SH2D1A* encoding SAP result in failure of T cell: B-cell interactions and inability to propagate 2B4- and NTBA-mediated signals promoting cytotoxicity and instead favor an inflammatory cytokine storm that drives HLH (77, 80–84). Although XLP1 and APDS patients fail to control EBV infection, both patient cohorts harbor EBV-specific T cells and their CD8 T cells show normal *in vitro* effector functions in response to SAP-independent stimuli (82, 85). Interestingly, positive signaling for cytotoxicity induced by receptors of the SLAM family (e.g., 2B4 and NTBA) that utilize the SAP adaptor involves PI3K/AKT activity (86, 87). Thus, both APDS and XLP1 share the feature of EBV susceptibility; however, unlike XLP1 patients, APDS patients are not susceptible to HLH. We hypothesize that hyperactive PI3K T-cell intrinsically drives polyclonal senescence and prevents a cytokine storm and HLH by limiting homing, expansion, and survival of EBV-specific T cells, as described further below (Figure 1B). Indeed, T cells from APDS patients exhibit enhanced stimulation-induced apoptosis (10), which is a feature shared with patients deficient in the anti-apoptotic factor XIAP who are susceptible to EBV and HLH (88, 89). Poor survival of EBV-reactive T cells may be a common underlying feature of EBV susceptibility in both XIAP deficiency and APDS, although the HLH phenotype in XIAP deficiency is poorly understood (90, 91).

The PI3K-driven expansion of effector CD8 T cells in APDS (11, 14) raises the question of why they cannot control EBV infection. The answer might come from the differentiation state of CD8 T cells since peripheral blood T cells in APDS patients are terminally differentiated with characteristics of senescence (92) (Table 1), including low IL-2 secretion, shortened telomeres, and poor proliferative capacity. Studies in mouse tumor models have similarly shown that senescent T cells exhibit *in vivo* defects including reduced survival, proliferation, IL-2 production, lymphoid homing, and tumor rejection (Figure 1B) (93, 94). Replicative senescence occurs when telomere erosion that occurs with each cell division reaches a critical point, leading to irreversible cell-cycle arrest through activation of the DNA damage response that is thought to protect from cellular transformation by preventing genomic instability and infinite proliferation (95). CD8 and CD4 T-cell immunosenescence has been observed in elderly individuals (96), and numerous studies demonstrate a high correlation between T-cell aging and persistent infections (e.g., CMV, EBV and HIV) (97–99) or the development of tumors (100, 101). A closer look at CMV-specific T cells has revealed a link between aging and increased frequency of CMV-specific CD8 T cells with a senescent phenotype (102, 103), suggesting that chronic antigen stimulation might drive T-cell senescence. Consistent with this hypothesis, the expression of the telomerase reverse transcriptase (TERT) that regulates the length of telomeres drastically declines in CD8 T cells after repeated antigen stimulation and acquisition of a senescent phenotype (104). Interestingly, overexpression of TERT increases the proliferative capacity of stimulated T cells (105), and using a pharmacological activator of TERT enhances CD8 T-cell-mediated control HIV infection *in vitro* (106).

Thus, immunosenescence represents a plausible contributor to defective EBV control in APDS patients, as CD8 T cells might not be able to clonally expand and mount a robust and specific response against EBV despite their prominent effector phenotype (11). While repeated EBV antigen stimulation seems to be an attractive hypothesis for driving T-cell immunosenescence in APDS, patients without active herpesviruses still have a high frequency of senescent T cells (Table 1), indicating that immunosenescence is likely not restricted to antigen-specific T cells. Instead, the hyperactivation of PI3K, a signaling pathway known to play multiple roles in survival, metabolism, cell growth, and cell-cycle progression (107–109), likely drives senescence by promoting exuberant *in vivo* CD8 T-cell proliferation (and resulting in clinical features of lymphoproliferation). Moreover, several studies have linked increased PI3K/AKT/mTORC1 activity with senescence in immortalized and primary cells (110–115). Interestingly, studies in cells with hyperactive PI3K signaling or mTORC1 inhibition with rapamycin have led to a model in which PI3K/AKT/mTORC1 signaling plays an early role in cell senescence induction without hyperproliferation as a prerequisite (110). While this latter set of data suggests that DNA damage is not a driving factor for PI3K-dependent senescence, other studies further proposed that PI3K/AKT contributes to reactive oxygen species production to cause irreparable chromosomal damage and irreversible cell-cycle arrest (111, 116). Although it is clear that the PI3K pathway plays an important role in senescence, further investigation is required to fully understand senescence of CD8 T cells in APDS patients. As such, APDS provides an invaluable opportunity to study immunosenescence and roles for PI3K in its regulation in humans.

Thus, hyperactive PI3Kδ may drive CD8 T-cell growth, terminal differentiation, and immunosenescence, although the detailed molecular basis of T-cell senescence in APDS patients remains to be fully elucidated. This state is associated with altered CD8 T-cell functions, including decreased proliferation and increased TCR restimulation-induced cell death, that might contribute to failure of APDS patients to adequately control EBV.

## Conclusion

Genomics has greatly advanced studies of PIDs (117, 118), shedding light on genes critical for human immunity. The recently solved PID called APDS highlights important roles for regulated PI3Kδ signaling in control of EBV through effects on B- and T-cell development and function.

## Author Contributions

JMC and CL prepared and wrote the minireview manuscript.

## Conflict of Interest Statement

The authors declare that the research was conducted in the absence of any commercial or financial relationships that could be construed as a potential conflict of interest.
